# James Smith Turner

**Published:** 1904-07

**Authors:** 


### Obituary.

### JAMES SMITH TURNER.

DENTISTRY knows no country, nor is it possible for it to lose interest in those not immediately connected with its local work. American dentists to-day mourn with our English brethren the loss of one of the most faithful exemplars of the best in our profession. For this, J. Smith Turner's name has become a household word among English-speaking dentists the world over. His death, therefore, at threescore years and ten, must be regarded as something more than a national loss. His activity up to the period of his last illness was seemingly equal to that of any former period of his life.

James Smith Turner belonged to a race of men who, having formed a standard of character, endeavor to live up to it, and no exertion was too great to have other men realize that the standard of ethics he had formulated for himself was the one to meet the highest needs of his profession. As the *British Dental Journal* says of him, "He was a strong man, raised up to fight a long and stubborn battle; he was always fighting, always winning in the end. . . . He always thought of the cause, and never of himself, and how he wore himself out to win not recognition, honors, wealth, or even professional success, but a solid, honorable, legalized status for the profession to which he belonged."

Such was the man, unselfish, devoted, sacrificing the good things of life that dentistry in Great Britain might occupy worthily a place among the honored professions.

James Smith Turner passed away from his earthly activities on Monday, February 22, 1904, and the interment took place at St. George's Cemetery, Ealing.

He was born at Edinburgh in 1832, and received his education in that city. He was apprenticed to an Edinburgh dentist by the name of Mien, and when he reached his twenty-first year he removed to London. This was in 1853. In 1857 he became a member of the College of Dentists. In 1863 he passed the necessary examinations and became an M.R.C.S. and an L.D.S. He was for forty-five years connected with the Middlesex Hospital. He delivered lectures there on dental surgery and acted as Dental Surgeon, and for many years was Consulting Surgeon to the Hospital.

He was subsequently elected to fill the position of Lecturer on Dental Mechanics in the London School of Dental Surgery. This position he held until 1879.

On July 26, 1880, the dental profession presented Mr. Tomes a portrait of himself and to Mr. Turner an inscribed clock and a purse of money.

In 1888 Mr. Turner was actively engaged in the management of the Dental Hospital of London.

He was a member of the "Odontological Society." The British Dental Association, in which he was an ever active member and later its President, presented Mr. Turner, in 1890, with his portrait, and a replica was also presented to Mrs. J. Smith Turner.

These facts, culled from the *British Dental Journal,* simply show the high appreciation in which J. Smith Turner was held by his colleagues, but they do not show the great work performed by this man. His labor was unceasing to advance dentistry in Great Britain. This was markedly in evidence in the labor he gave to the passing of the "Dentists' Act." "For weeks, months, years, night and day, the business was in hand. Parliamentary committees had to be attended and the House of Commons frequented night after night."

This was the man that the dentistry of Great Britain honors in memory to-day, and the dentists of the United States join with their brethren there in mourning the fact that one of the few laborers in the ranks who have unselfishly worked that the profession might live has passed away to be known no more among men. The writer of this never knew J. Smith Turner personally. That fact is of no material consequence. The spirit that actuated this man has no need of personal recognition. It permeates space and becomes part of the moral force of the universe, recognized everywhere by those energized by the same power. It will live, and J. Smith Turner, though dead, will rouse up others in all lands to go forward in a crusade against the commercial, selfish spirit that has gradually attempted the destruction of the very heart of professional life. It is such as he that has made the dentistry of Great Britain a model for the best in the United States to copy. May the dentists of this land take seriously to heart the life of this man. It is the one great lesson needed here. The golden age is measured by many, not from the results following a high ethical standard, but by that which glitters for a season. That which is born to live must be that which the J. Smith Turners of all lands and of all ages have lain down their lives to bring to perfection.

